# Modulation of the Immune Response to Respiratory Viruses by Vitamin D

**DOI:** 10.3390/nu7064240

**Published:** 2015-05-29

**Authors:** Claire L. Greiller, Adrian R. Martineau

**Affiliations:** Barts and the London School of Medicine and Dentistry, Queen Mary University of London, London E1 2AB, UK

**Keywords:** vitamin D, respiratory viruses, antiviral immunity

## Abstract

Background: Vitamin D deficiency has been shown to be independently associated with increased risk of viral acute respiratory infection (ARI) in a number of observational studies, and meta-analysis of clinical trials of vitamin D supplementation for prevention of ARI has demonstrated protective effects. Several cellular studies have investigated the effects of vitamin D metabolites on immune responses to respiratory viruses, but syntheses of these reports are lacking. Scope: In this article, we review the literature reporting results of *in vitro* experiments investigating immunomodulatory actions of vitamin D metabolites in human respiratory epithelial cells infected with respiratory viruses. Key findings: Vitamin D metabolites do not consistently influence replication or clearance of rhinovirus, respiratory syncytial virus (RSV) or influenza A virus in human respiratory epithelial cell culture, although they do modulate expression and secretion of type 1 interferon, chemokines including CXCL8 and CXCL10 and pro-inflammatory cytokines, such as TNF and IL-6. Future research: More studies are needed to clarify the effects of vitamin D metabolites on respiratory virus-induced expression of cell surface markers mediating viral entry and bacterial adhesion to respiratory epithelial cells.

## 1. Introduction

Viral acute respiratory infections (ARI) are a leading cause of morbidity and mortality worldwide, and, as such, are a major global health problem. They are also responsible for a huge economic burden, precipitating considerable absence from work and school, and large numbers of visits to clinicians.

The viral pathogens most commonly associated with acute respiratory infections are picornaviruses (including the species rhinovirus (RV) A, RVB, RVC and the enteroviruses A–D), orthomyxoviruses (influenza A, influenza B and influenza C), paramyxoviruses (including the parainfluenza viruses (PIV) 1–4, respiratory syncytial virus (RSV) and human metapneumovirus (hMPV)), coronaviruses, adenoviruses and human bocavirus (HBoV) [1,2,3,4,5]. The only routinely administered vaccine for a viral respiratory pathogen in the UK is against seasonal influenza: this is not 100% effective and may offer varying levels of protection against influenza infection in different seasons and different age groups [6]. The availability of effective antiviral drugs is also limited, with the effectiveness of approved influenza treatments, such as Oseltamivir (Tamiflu), debated [7,8,9], and no clinical treatments for common respiratory viruses such as rhinoviruses. New agents to prevent and treat viral respiratory infection are needed: the WHO initiative BRaVe (Battle against Respiratory Viruses) has recently been established to aid in the goal of developing new pharmacologic interventions to prevent and treat respiratory viruses [10].

Vitamin D is an immunomodulatory micronutrient [11,12]. As reviewed elsewhere [13], a number of observational studies have been carried out to determine the relationship between vitamin D status and acute respiratory viral infections, with the majority demonstrating independent associations between low vitamin D status and increased risk of acute viral respiratory infections. Clinical trials of vitamin D supplementation for the prevention of ARI have yielded heterogeneous results [14,15,16,17,18,19,20,21,22,23,24,25,26,27], with meta-analysis reporting moderate protective effects overall [28].

A number of *in vitro* studies have investigated the effects of vitamin D metabolites on host immune responses to respiratory viruses, but recent syntheses of this literature are lacking. In order to review these studies the PubMed database was searched using the terms “vitamin D” with the following respiratory viruses (rhinovirus, RSV, influenza, parainfluenza, human metapneumovirus, coronavirus, adenovirus, enterovirus and human bocavirus) to ensure a systematic review of the available literature. Inclusion criteria were studies which provided *in vitro* evidence as opposed to solely clinical studies, the availability of the full text, and for virus genera, such as enterovirus, which can infect multiple sites, the use of species or serotypes specifically associated with respiratory disease.

## 2. The Host Immune Response to Viral Respiratory Infection

### 2.1. Innate Immune Response

When a respiratory virus is inhaled it first binds to non-specific receptors on the respiratory epithelium, usually glycolipids or glycoproteins such as intercellular adhesion molecule (ICAM)-1. Membrane fusion or endocytosis follows, thus internalizing the virus and enabling subsequent replication, transcription and translation of new viruses which can then be released to infect new cells. However, once a cell has been infected, pathogen-associated molecular patterns (PAMPs) on the virus can be recognised by various intracellular innate pathogen recognition receptors (PRRs) such as the toll-like receptors (TLRs), retinoic-acid-inducible gene-I (RIG-I)-like receptors (RLRs) and nucleotide binding-oligomerisation domain (NOD)-like receptors (NLRs). Pulmonary epithelial cells have been shown to express all of the known human TLRs and RLRs which detect viruses, and ligands for these PRRs activate epithelial cells in order to initiate a rapid immune response against viral invasion [29]. In addition to direct infection of epithelial cells, intraepithelial dendritic cells (DCs), DCs residing just below the respiratory epithelium, and tissue-resident macrophages continually sample particles in the airway lumen and can internalize them by phagocytosis and macropinocytosis, thus activating PRRs and initiating an immune response [30,31].

**Figure 1:**
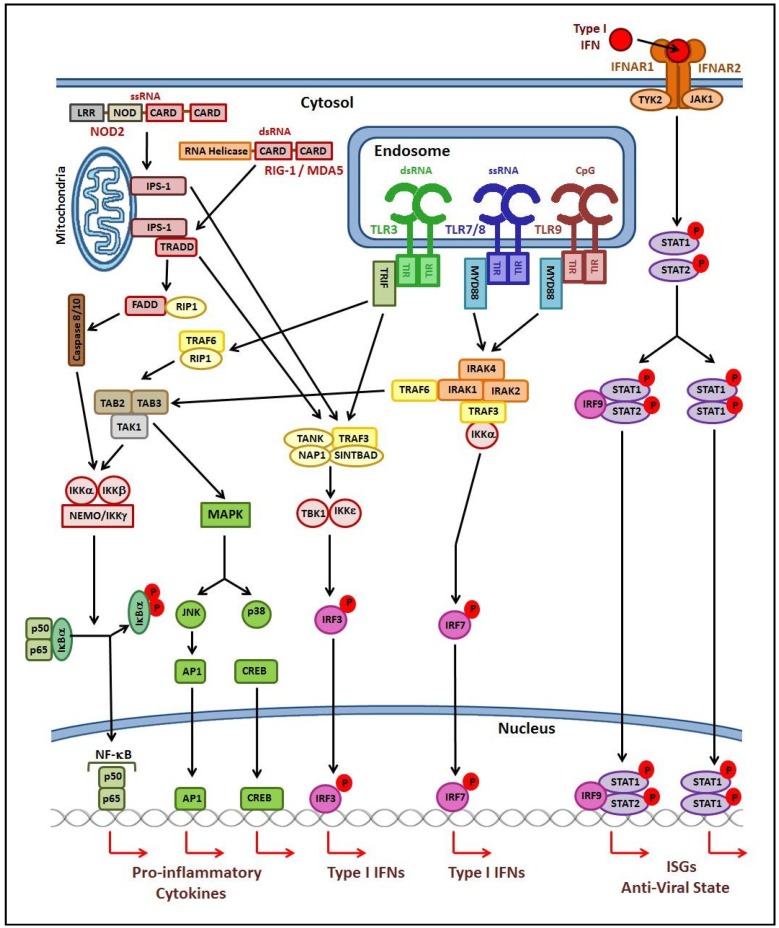
Pathogen recognition receptor signalling following viral infection. Ligand-induced dimerisation occurs following PAMP recognition by endosomal TLRs, which engages the Toll-IL-1 receptor (TIR) domains to initiate adaptor molecule recruitment and signal transduction. MYD88-dependent signalling results in the formation of an IRAK/TRAF6 complex, which phosphorylates IRF7 to initiate transcription of type I IFN genes, and activates a TAK1/TAB2/3 complex to drive transcription of pro-inflammatory cytokine genes via activation of NF-κB, AP1 and CREB. TRIF-dependent signalling can also activate NF-κB, AP-1 and CREB via recruitment of TRAF6 and RIP1. Alternatively, TRAF3 is recruited, resulting in phosphorylation of IRF3 which translocates into the nucleus to induce expression of type I IFNs. RIG-I and MDA5 are also able to activate NF-κB and IRF3 via interaction with IPS-1 localized on the mitochondrial membrane through homophilic interactions between their CARD domains. Similarly, CARD domains of NOD2 also interact with IPS-1 resulting in transcription of type I IFN genes. The type I IFNs produced bind to their receptor, and, via STAT-mediating signalling, initiate gene transcription.

The intracellular TLRs 3, 7, 8 and 9 are mainly located on the endoplasmic reticulum (ER) membrane before UNC93B1-dependent (an ER multi-transmembrane-domain-containing protein) trafficking to the endolysosome following viral infection [32,33]. These nucleic acid-sensing TLRs recognise single-stranded RNA (TLR 7/8) or unmethylated CpG double-stranded DNA motifs (TLR 9) of the viral genome, or the intermediary double-stranded RNA (TLR 3) produced during viral replication [34,35,36,37]. Additionally, TLR4 and TLR2 receptor complexes are able to traffic to the endolysosome and may play a role in viral recognition [37,38,39,40,41]. Viruses which avoid recognition by TLRs, can be recognised by RLRs which are present throughout the cytosol, with RIG-I important in the immune response to many RNA viruses [42], and melanoma differentiation-associated gene 5 (MDA5) crucial in the recognition of picornaviruses [43]. Additionally, the cytosolic NLR NOD2, whilst normally associated with the recognition of bacterial muramyl dipeptide, has also been demonstrated to be involved in the recognition of the ssRNA genome of RSV [44]. Despite differences in viral genomes, replication strategies, and the types of PRRs activated, common signalling pathways are utilized. Thus recognition of viral pathogens elicit conserved outcomes, with the interferon regulatory factor (IRF)-mediated production of type I IFNs a central feature, along with nuclear factor kappa B (NF-κB)- and mitogen-activated protein kinase (MAPK)- mediated regulation of various inflammatory cytokines [37,45] (Figure 1).

Upon PAMP-PRR interaction and the activation of signalling transduction pathways, the type I IFNs (IFN-α and IFN-β) are some of the earliest cytokines to be produced. Their transcription and subsequent binding to the IFN receptor induces expression of a variety of interferon-stimulated genes (ISGs), with their products altering antiviral and immunomodulatory actions to limit and clear infection [45]. Features of the induced anti-viral state include resistance to viral replication in all cells, induction of apoptotic cell death in infected cells, increased major histocompatibility complex (MHC) class I expression to enhance antigen presentation, activation of dendritic cells (DCs) and macrophages, and stimulation of natural killer (NK) cells to enhance their cytolytic activity [46]. The inflammatory cytokines TNF-α, IL-1β, IL-6 and IL-12 are also produced at an early stage of the innate immune response. These cytokines promote leukocyte extravasation by increasing endothelial expression of adhesion molecules, such as ICAM-1 and VCAM-1, increase vascular permeability, induce synthesis of acute phase proteins, and contribute to recruitment and activation of cells of the adaptive immune response. Additionally, IL-1β and TNF-α amplify the inflammatory response by triggering further NF-κB and MAPK activation [47].

Innate PRR signalling also results in the production of the chemokines CXCL8, CXCL9, CXCL10 and IL-15 which facilitate recruitment of neutrophils and NK cells, respectively [48,49]. While neutrophils and macrophages have well-defined roles in bacterial innate immunity, their function in antiviral immunity is less clear and is likely to be minimal, with inhibition of neutrophil recruitment to the lung demonstrated to have no effect on the course of influenza infection [50], and the antigen presenting capacity of macrophages in the respiratory tract demonstrated to be limited [51,52]. The antimicrobial peptide LL-37, which is produced by both neutrophils and macrophages, is traditionally viewed as a component of the immune response to bacteria. However, anti-viral activity of LL-37 has also been demonstrated against both enveloped and non-enveloped viruses, including influenza virus and RSV [53]. It has been shown to disrupt the membrane of influenza virus [54], modify cytokine production [54], enhance TLR3 signalling [55], and have direct effects on RSV viral particles and infected epithelial cells, with diminished spread of infection and inhibited production of new virus particles [56]. Macrophages may also have a critical role in the resolution of inflammation and clearance of infection to prevent immunopathology. They are able to phagocytose infected cells and apoptotic cellular debris, which, if left in the lumen, can release chemokines, such as CXCL9 and CXCL10, resulting in recruitment of inflammatory cells and increased bystander tissue damage. In the absence of alveolar macrophages, or when macrophages have impaired function, airway occlusion can occur following RSV infection due to the accumulation of inflammatory cells, virus-infected cells, apoptotic debris and serum proteins, resulting in severe and potentially fatal bronchiolitis [57]. Similarly, following influenza infection in mice devoid of alveolar macrophages, pulmonary alveolar proteinosis (PAP) was demonstrated owing to the accumulation of surfactant material and a failure in efferocytosis, causing impaired gas exchange and fatal hypoxia [58]. Therefore, macrophages appear to have a vital role in the antiviral immune response, by producing antimicrobial peptides during the innate response, and phagocytosing dead cells and cellular debris to prevent immunopathology.

### 2.2. Adaptive Immune Response

PRR signalling facilitates the maturation and trafficking of dendritic cells, with the release of the chemokines CCL2 and CCL20, and increased expression of CCR7 [59,60]. Thus, around 72 h after infection, DCs with antigen-MHC complexes migrate through the afferent lymph vessels to secondary lymph nodes where they form interactions with naive CD4^+^ and CD8^+^ T lymphocytes. These T-lymphocytes are activated, proliferate, differentiate into effector T-cells and migrate via efferent lymph vessels into the circulation. Multiple chemokines, such as CCL3, CXCL9 and CXCL10, are expressed in the respiratory epithelium and result in changes in integrin affinity, allowing effector T-cells to bind to the endothelium and migrate into the infected tissue [37,61,62,63,64]. For efficient and effective viral clearance Th1 effector T-cells are required, which produce IL-2, TNF-α and IFN-γ to activate NK cells and induce generation of cytolytic molecules. CD8^+^ effector T-cells and NK cells can then induce apoptosis of infected cells via the release of cytolytic granules or by direct interaction between surface Fas receptor and Fas ligand (FasL) [37]. B-cells have also been demonstrated to play an important role in the immune response to highly pathogenic viral infections. Contact between CD4^+^ T-cells and naive B-cells in secondary lymphoid tissues results in their proliferation and antibody class-switching, with neutralizing virus-specific antibodies crucial for optimal viral clearance. Additionally, viral components expressed on infected cells allow antibodies to bind, thus initiating antibody-dependent cell-mediated cytotoxicity (ADCC), whereby CD16 on NK cells recognises the Fc portion of antibodies bound to the surface and kills the target cell [37,65,66,67]. Complement is also increasingly being recognised as an important component of anti-viral immunity, as reviewed elsewhere [68]. The evolution of evasion mechanisms by viruses emphasises the importance of complement in anti-viral immunity, with, for example, the matrix protein M1 of influenza A able to bind to C1q to prevent interaction with antibodies on the surface of infected cells [68,69]. Therefore, combined, these effector mechanisms of the adaptive immune response rapidly clear the viral infection.

## 3. Immunomodulatory Actions of Vitamin D Metabolites

**Figure 2:**
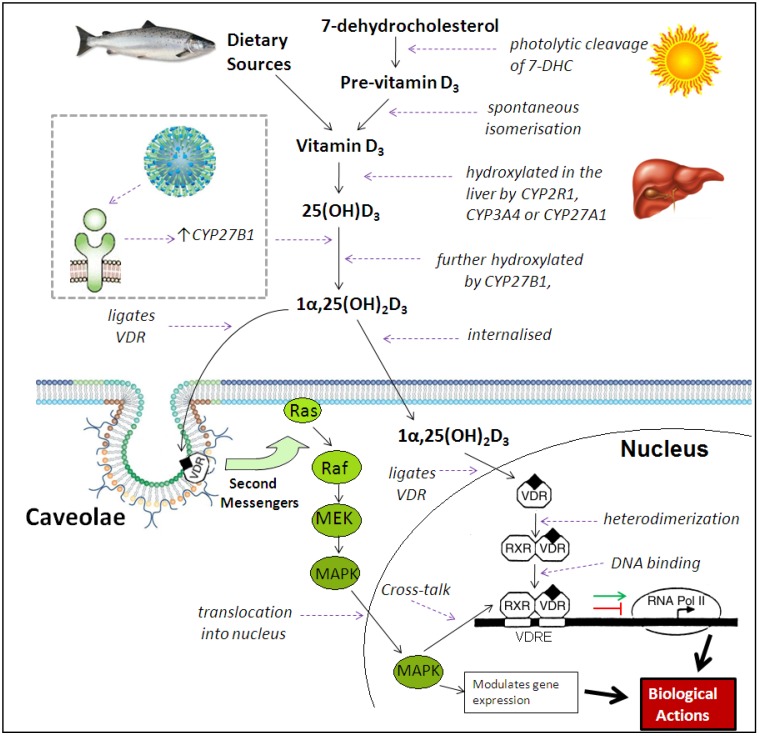
Metabolism of 1α,25(OH)_2_D_3_. Vitamin D_3_ is either obtained from dietary sources or UV synthesis, before two hydroxylations occur to produce the active metabolite 1α,25(OH)_2_D_3_. TLR ligation can also increase levels of CYP27B1, resulting in enhanced 1α-hydroxylation of 25(OH)D_3_. The 1α,25(OH)_2_D_3_ then binds to nuclear or membrane vitamin D receptors (VDRs). Nuclear VDR ligation results in heterodimerization with retinoid X receptor (RXR) and binding to vitamin D responsive elements (VDRE) in promoter regions of responsive genes. Components of the RNA polymerase II complex are then recruited for induction of gene transcription, or transcription is repressed. Membrane caveolae-associated VDR ligation results in the activation of second messenger systems, with one effect being the initiation of Ras/MAPK signal transduction. Nuclear MAPK modulates gene expression and engages in cross-talk with the VDR-RXR-VDRE complex. (Adapted from Slatopolsky *et al.* [104], and Parton and Simons [105]).

Vitamin D (cholecalciferol) is acquired from dietary sources, primarily oily fish, or from UV-mediated synthesis in the skin, before it is metabolised in the liver to form 25(OH)D_3_. This 25(OH)D_3_ is the circulating form of vitamin D, with a half-life estimated to range from two weeks to two months [70,71,72], and its serum concentration is used to define vitamin D status. Further hydroxylation results in the synthesis of 1α,25(OH)_2_D_3_ and ligation of nuclear vitamin D receptors (VDRs), allowing subsequent binding to vitamin D responsive elements (VDREs) in promoter regions of specific genes, resulting in the repression or induction of gene transcription [11] (Figure 2). VDRs have also been shown to be located within the plasma membrane or its caveolae components [73,74,75], with ligation resulting in 1α,25(OH)_2_D_3_-mediated non-genomic rapid responses via the activation of second messenger systems, such as phospholipase C, protein kinase C and phosphatidylinositol-3′-kinase (PI3K), thus initiating various intracellular effects, such as the opening of voltage-gated calcium channels and Ras/MAPK signal transduction [75,76]. The detection of VDR abundance throughout the immune system, the observation that VDR expression is regulated by immune signalling, and the detection of CYP27B1 expression by immune cells has directed vitamin D research into the area of immunology [77,78,79,80,81], with associations of low serum 25(OH)D_3_ levels seen with autoimmune diseases, cancer, cardiovascular disease and respiratory infections [82,83,84,85].

Significantly, a link has been made between TLR ligation and cytokine secretion, the expression of CYP27B1 and the expression of the VDR. In the absence of any influence by calcium homeostatic agents [86,87,88], and with a defective negative feedback mechanism [89,90], extra-renal 1α-hydroxylation in cells such as monocytes and macrophages is regulated primarily by immune inputs. Therefore, this renders the immune system responsive to circulating levels of 25(OH)D and allows the sustained and potentially beneficial production of 1,25(OH)_2_D during an immune response [90].

Vitamin D metabolism in macrophages is linked to pathogen recognition, thus making it an integral part of the innate immune response [91]. Ligation of the TLR2/1 heterodimer in macrophages has been demonstrated to up-regulate CYP27B1, and the TLR8 ligands CL097 and ssRNA40 have also both been demonstrated to induce a dose-dependent increase in CYP27B1 mRNA and protein expression in macrophages [92]. Similarly, ligation of TLR4 by LPS up-regulated CYP27B1 expression in monocytes [93,94]. In dendritic cells, ligation of TLR4 by LPS or monophosphoryl lipid A (MPLA), and ligation of TLR3 by polyI:C induced expression of CYP27B1, altering DC migratory properties to allow their localisation into non-draining secondary lymphoid organs to present antigen peptides to CD4^+^ T-cells [79,95,96,97]. Similarly, in human tracheobronchial epithelial (hTBE) cells, polyI:C stimulation and addition of live RSV were demonstrated to increase CYP27B1 expression, enhance 25(OH)D conversion to 1,25(OH)_2_D, and amplify cathelicidin mRNA [98]. With the expression of TLRs in multiple cell types, and the ability to respond to a variety of pathogens, it is also possible that other TLRs or alternate PRRs may promote extra-renal expression of CYP27B1, allowing locally generated 1,25(OH)_2_D to have even more extensive effects on the immune response.

The exact mechanism by which TLR ligation enhances CYP27B1 production has not been fully defined, although the JAK-STAT, NF-κB and p38 MAPK pathways, and phosphorylation of the transcription factor C/EBPβ by p38 MAPK have been implicated [94]. Additionally, IL-15 has been demonstrated to act as an intermediary in promoting localized activity of CYP27B1 and synthesis of 1,25(OH)_2_D. TLR 2/1 ligation by a triacylated lipopeptide of the *M.tuberculosis* 19 kDa antigen in human monocytes induced IL-15 secretion, which was required for the upregulation of CYP27B1 and the VDR, and subsequent downstream production of LL-37 [99]. Other cytokines, such as IL-13, IFNγ, IL-4, IL-1, IL-2 and TNF-α, have also been implicated as regulators of CYP27B1 expression and vitamin D metabolism [87,94,100,101,102,103]. The pathways involved in cytokine-regulated transcription of CYP27B1 are likely the same ones as utilized by TLR ligation-induced regulation, as described above [101].

Therefore, extra-renal 1,25(OH)_2_D synthesis has been shown to be regulated by TLR ligation and cytokine secretion, utilizing an intricate cross-talk between various signalling pathways. As such, in the presence of sufficient circulating levels of 25(OH)D, infection by respiratory viruses resulting in recognition by TLRs and cytokine production is able to increase levels of 1,25(OH)_2_D, hypothetically altering the immune response to better respond to these pathogens.

## 4. Effects of Vitamin D on the Innate Immune Response to Respiratory Pathogens

Vitamin D has diverse and extensive effects on the immune system, due to the expression of the VDR and the enzyme CYP27B1 by most immune cells [11,77,81,106]. The most frequently demonstrated effect of vitamin D is that on LL-37, and, as such, its expression is commonly used to demonstrate increased CYP27B1 expression and 1,25(OH)_2_D activity. Cathelicidins are multifunctional antimicrobial peptides, and the sole form in humans is Human Cationic Antimicrobial Peptide of 18KDa (hCAP-18). From this precursor, the active form of LL-37 is cleaved by serine protease 3 in neutrophils [107]. While the method of cleavage has not been demonstrated in other cells types, LL-37 has been shown to be present in epithelial cells, monocytes, NK cells, B-cells and γδ T-cells [56,108], and can be secreted by respiratory epithelial cells onto the airway surface to form a first line of defence against invading pathogens [109]. It has many functions, as described elsewhere [110,111,112,113,114,115], such as direct facilitation of chemotaxis of immune cells and modification of DC differentiation, and, while traditionally viewed as a component of only the immune response to bacteria, has also been shown to act directly against viruses [53,54,56]. Numerous studies have demonstrated the role the 1,25(OH)_2_D-ligated VDR plays in binding to a VDRE in the promoter of the cathelicidin gene to enhance hCAP-18 production [116,117,118,119], thus suggesting a potential mechanism by which vitamin D may enhance innate immunity to respiratory infections.

The proximal promoter region of DEFB4 gene also contains a VDRE, allowing 1,25(OH)_2_D to upregulate expression of β-defensin 2 [116]. This is another antimicrobial peptide, which, similarly to LL-37, is able to induce chemotaxis of immune cells and has been shown to inhibit RSV infection [120,121].

In addition to antimicrobial peptide induction, vitamin D has been demonstrated to modulate the innate immune system in a variety of other ways. Monocyte differentiation into macrophages is induced [122], with enhancement of the phagocytic and chemotactic capacity of macrophages [11,123], thus facilitating efferocytosis and preventing immunopathology. One of the signalling pathways regulated by vitamin D is the class III phosphatidylinositol 3-kinase complex (PI3KC3), with PI3K signalling associated with monocyte and macrophage generation of ROS and iNOS [124,125]. The oxidative burst has been demonstrated to have beneficial antiviral effects [126,127,128,129], although aberrant induction is associated with pathophysiology and tissue damage [130,131,132]. As such, vitamin D may have an important role in redox homeostasis, with evidence of both pro-oxidative induction of ROS and iNOS to boost the antiviral response [125,133], and antioxidative inhibition of iNOS and induction of ROS scavenging pathways to prevent immunopathology [134,135,136]. The ability of 1,25(OH)_2_D to induce monocyte autophagy has also been demonstrated. Autophagy acts as part of the immune system to remove damaged proteins and organelles, and is an important host defence mechanism against viral infections [137]. Vitamin D has been shown to induce autophagy by regulating multiple associated pathways, such as Bcl-2, mammalian target of rapamycin (mTOR), class III phosphatidylinositol 3-kinase complex, and cathelicidin production, thus potentially enhancing clearance of viruses and viral components [124,138,139].

Pattern recognition receptors have also been demonstrated to be regulated by vitamin D. Expression of TLR2 and TLR4 is inhibited in monocytes, resulting in impaired downstream signalling and hyporesponsiveness to PAMPS. With the observation that this effect is most prominent after 72 h, a negative feedback mechanism has been suggested, whereby excessive TLR activation is prevented at later stages of infection to dampen inflammation [11,140]. Conversely, CD14, an accessory protein to TLR4 which has also been linked to TLR2 [141], was up-regulated by 1,25(OH)_2_D [140,142,143], but this effect was not sufficient to restore downstream TLR signalling [140]. Additionally, as opposed to its effects in monocytes, 1,25(OH)_2_D has been demonstrated to induce up-regulation of TLR2 in keratinocytes, allowing a mechanism by which vitamin D may prevent infection of wounds [144]. Finally, the intracellular receptor NOD2 is induced by 1,25(OH)_2_D in myeloid and epithelial cells, via two distal VDREs in the *NOD2* gene. Addition of MDP (a lysosomal breakdown product of bacterial peptidoglycan [11]) to 1,25(OH)_2_D-induced NOD2 enhanced NF-κB signalling and subsequent β-defensin 2 expression [145].

## 5. Effects of Vitamin D on the Adaptive Immune Response to Respiratory Pathogens

Vitamin D also modulates the adaptive immune response, and acts as a key intermediary between innate and adaptive immunity due to its influence on antigen presentation.

Dendritic cells are the most potent antigen-presenting cells, and, as such, have a direct effect on lymphocyte activation and induction of the adaptive immune response. They reside in peripheral tissues in an immature state, sampling the environment and mediating antigen uptake, until a maturation signal induces migration to local lymph nodes and subsequent T-cell activation. Addition of 1,25(OH)_2_D has been demonstrated to inhibit DC differentiation, maturation and antigen presentation, with an associated decrease in markers such as CD1a, MHC class II, and the co-stimulatory molecules CD40, CD80 and CD86 [146,147,148], as well as abrogating the chemotactic response to CCL4 and CCL19 [149]. Already differentiated dendritic cells can also be redirected back towards a monocytic phenotype by the restoration of the monocytic marker CD14 [147,150].

The antigen-presenting and T-cell stimulatory capacity of monocytes and macrophages is also impaired by 1,25(OH)_2_D, with a decrease in MHC class II, CD40, CD80 and CD86. IL-12 production is suppressed in both activated DCs and macrophages, due to the 1,25(OH)_2_D-mediated down-regulation of NF-κB activation [151], while influence on the expression of TNF-α is dependent on the differentiation state of the cells, with a reduction observed following 1,25(OH)_2_D administration in LPS-stimulated monocytes and PBMCs [140,152].

The main function of DCs is to initiate T-cell responses, and thus the effect of 1,25(OH)_2_D on DCs has a major impact on T-cells. The decreased surface expression on DCs of co-stimulatory molecules and MHC class II results in a tolerogenic phenotype, with DC production of IL-12 (which is involved in driving Th1 differentiation) and IL-23 (which is involved in driving TH17 differentiation) inhibited by 1,25(OH)_2_D [11,150]. Even when cultured with committed T-cells, these tolerogenic DCs caused hyporesponsiveness, decreased T-cell proliferation and reduced IFN-γ secretion [146,153]. IL-12, as well as stimulating the development of Th1 T-cells, also inhibits the development of Th2 cells, thus resulting in vitamin D shifting the balance of T-cells from a Th1 to a Th2 phenotype [154]. Concomitantly, DC production of IL-10 is increased. IL-10 is a cytokine with pleiotropic effects in immunoregulation, and levels in BAL fluid have been shown to be inversely correlated with severity/incidence of asthma [155,156]. This IL-10 production drives development of regulatory T-cells (Tregs), and these Tregs are able to secrete more IL-10 as well as the immunomodulatory cytokine TGF-β, while release of the Treg cytokine CCL22 is also increased [11]. Th17 cells and IL-17, have also been shown to be decreased, with calcitriol (1,25(OH)_2_D) reducing IL-17 production in a mouse colitis model and impairing commitment to the Th17 lineage in mice with experimental autoimmune uveitis [157,158], although these effects may not translate to the human respiratory system. Th17 cells, by releasing IL-17, initiate an inflammatory response dominated by neutrophils. While high levels of IL-17 production are associated with chronic inflammation and severe immunopathology [159], deficient levels, as seen in Hyper-immunoglobulin E syndrome (HIES), result in recurrent fungal and bacterial infections [160]. Therefore, vitamin D may have a beneficial role in attenuating immunopathology caused by some infections, but be detrimental in other fungal and bacterial infections.

1,25(OH)_2_D has also been demonstrated to have direct effects on T-cells, independent of DC activity. While the role of DCs in the induction of Tregs has been described, it has also been shown that 1,25(OH)_2_D in combination with dexamethasone can induce a Treg population in the absence of APCs [161]. The proliferation and cytokine profiles of T-cells are also directly altered by 1,25(OH)_2_D. Production of IL-2, IFN-γ, TNF-α, IL-17 and IL-21 are all inhibited [157,162,163], with inhibition of IFN-γ further precluding macrophage activation, thus attenuating antigen presentation and the recruitment of other T-cells [164]. This direct inhibition of Th1-priming cytokines further skews T-cell differentiation towards a Th2 phenotype. 1,25(OH)_2_D is also able to upregulate the Th2-specific transcription factors GATA-3 and c-maf, resulting in increased production of IL-4, IL-5 and IL-10 [165]. B-cells are also affected by vitamin D, with modulation of T-cell responses altering the B-cell compartment, as well as having direct effects on B-cells themselves [11]. 1,25(OH)_2_D is able to inhibit proliferation, plasma-cell differentiation, immunoglobulin secretion and memory B-cell generation, while inducing B-cell apoptosis [80]. As such, vitamin D supplementation has been used in the treatment of B-cell-associated autoimmune diseases such as systemic lupus erythematous [166,167]. Finally, it has been suggested that vitamin D may affect other lymphocyte subsets, with VDR-KO mice presenting with fewer invariant natural killer (iNKT) cells [168], and CD8^+^ T-cells from MS patients secreting less IFN-γ and TNF-α and more IL-5 and TGF-β following 1,25(OH)_2_D treatment [169].

However, the mechanisms behind any potential beneficial role of vitamin D are unclear, with conflicting cellular studies on the effects of vitamin D on Th2 cells [170,171], with both enhancement [165] and inhibition [172] of IL-4 synthesis demonstrated. 1,25(OH)_2_D has also been shown to down-regulate DC-derived Ox40L, which is required for Th2 priming, thus, resulting in a reduced Th2 cytokine response in CD4^+^ T-cells from patients with allergic bronchopulmonary aspergillosis [173], thus contradicting evidence that vitamin D skews the T-cell phenotype towards a Th2 one. Additionally, the decrease in Th1 immunity which has been observed [11] would suggest a diminished immune response to pathogens, contrasting to the evidence suggesting an improved response to respiratory tract infections after vitamin D supplementation. Finally, while studies have demonstrated direct effects of 1,25(OH)_2_D administration on lymphocytes, others have shown that when using the inactive metabolite 25(OH)D, DCs are required to convert this precursor to the active 1,25(OH)_2_D to exert its immunomodulatory effects [174]. This indicates that administration of different vitamin D metabolites may result in a different response. Therefore, while vitamin D clearly acts as an immunomodulatory molecule with a wide range of effects demonstrated, the precise mechanisms are currently unclear, with the conflicting results reported also adding to the uncertainty of its actions.

**Figure 3:**
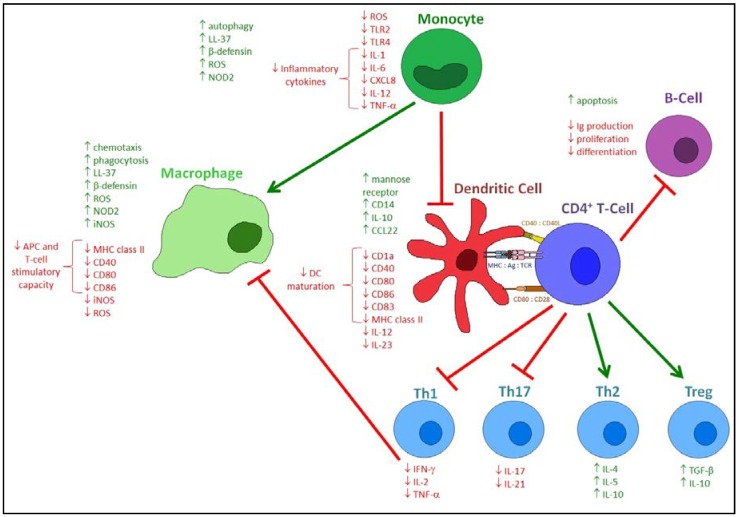
The immunomodulatory actions of 1,25(OH)_2_D. 1,25(OH)_2_D has diverse and extensive effects on the immune compartment. The innate immune response is affected, with monocytes producing more LL-37 and β-defensin, with increased NOD2 expression and autophagy, while also producing diminished amounts of inflammatory cytokines, with decreased expression of TLR2 and TLR4. Differentiation into macrophages is increased, with macrophages having an increased capacity for phagocytosis and chemotaxis. However, their APC and T-cell stimulatory capacity is decreased. Monocyte and macrophage production of ROS and iNOS is able to both be induced and inhibited, thus regulating their balance. Differentiation into DCs is inhibited, with DCs expressing decreased levels of maturation surface markers. DC production of IL-12 and IL-23 is decreased, while mannose receptor expression and production of IL-10 and CCL22 are increased. When these tolerogenic DCs interact with T-cells, development of Tregs and Th2 cells is increased, with increased production of IL-10, TGF-β, IL-4 and IL-5. The development of Th1 and Th17 cells is inhibited, with decreased production of IL-2, IFN-γ and TNF-α, and attenuation of macrophage activation. B-cells are also affected by 1,25(OH)_2_D, demonstrating decreased immunoglobulin production, proliferation and differentiation, but increased apoptosis.

The main immunomodulatory effects of vitamin D are summarised in Figure 3.

## 6. Effects of Vitamin D Metabolites on the Host Response to Respiratory Viruses

**Figure 4:**
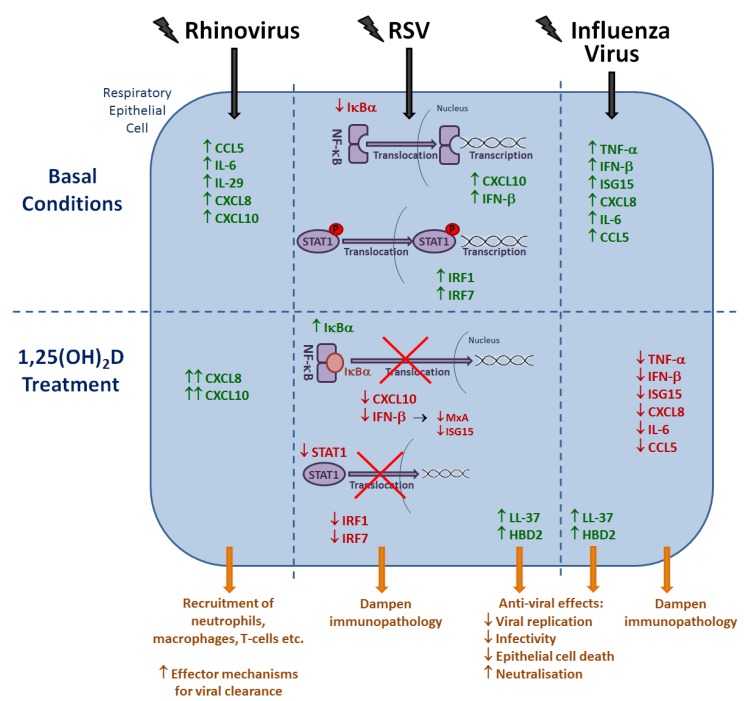
The immunomodulatory actions of 1,25(OH)_2_D against respiratory viruses. Rhinovirus infection of epithelial cells results in increased production and secretion of pro-inflammatory cytokines and chemokines, with the secretion of CXCL8 and CXCL10 further enhanced following treatment with 1,25(OH)_2_D. During RSV infection, IκBα expression is reduced, resulting in increased transcription of NF-κB-driven genes. STAT1 is also phosphorylated and able to translocate into the nucleus resulting in increased expression of IRF1 and IRF7. Pre-treatment with 1,25(OH)_2_D increases IκBα expression and decreases STAT1 phosphorylation, resulting in decreased production of CXCL10, IFN-β, MxA, ISG15, IRF1 and IRF7. Similarly, influenza A infection causes increased expression of pro-inflammatory cytokines and chemokines, with 1,25(OH)_2_D treatment causing decreased expression of TNF-α, IFN-β, ISG15, CXCL8, IL-6 and CCL5. Finally, 1,25(OH)_2_D is also able to increase LL-37 and HBD2 production, which have been shown to have antiviral effects against both RSV and influenza.

While a number of *in vitro* studies have described the general effects of vitamin D metabolites on the function of immune cells and secretion of inflammatory molecules, as described above, experiments investigating the specific antiviral effects of vitamin D on each respiratory virus are limited. The available literature is summarised below, and presented in Figure 4.

### 6.1. Rhinovirus

Rhinovirus is the most common aetiologic agent of the common cold, which in turn is the most frequent acute infection in the industrialised world, associated with two to three episodes per year in adults, and five to seven per year in children [175,176,177]. However, only one study has been carried out to determine the effect of vitamin D on rhinovirus infection [178]. Experiments were carried out in which primary human bronchial epithelial cells (hBECs) were treated with 25(OH)D or 1,25(OH)_2_D and subsequently infected with RV-16. Incubation with vitamin D metabolites had no effect on viral replication, and, whilst RV-16 infection induced hBEC secretion of CCL5, IL-6 and IL-29, incubation with vitamin D metabolites did not alter this effect. Treatment with vitamin D did, however, enhance secretion of CXCL8 and CXCL10, both in the presence and absence of rhinovirus infection. These molecules are pro-inflammatory chemokines, responsible for the recruitment of immune cells such as neutrophils, macrophages and T-cells to the site of infection, and could thus act as an effector mechanism as to how vitamin D alters the antiviral response to rhinovirus infection.

### 6.2. RSV

RSV is usually associated with illness in young children, with most infants having been infected by the age of three, causing a mild URI for the majority, but severe bronchiolitis in some [179,180]. It has also been associated with a higher risk of developing asthma in later life [181]. Vitamin D deficiency has been linked to RSV susceptibility, with low cord blood plasma concentrations of 25(OH)D related to RSV incidence in the first year of life [182], and single nucleotide polymorphisms (SNPs) in the VDR (rs10735810 and the *Fokl* SNP) and vitamin D binding protein (the GC1s variant) associated with a genetic predisposition to RSV bronchiolitis [183,184,185,186]. However, as with rhinovirus, *in vitro* work investigating the specific effects of vitamin D on the immune response to RSV is limited.

In a study using primary human tracheobronchial epithelial (hTBE) cells treated with 1,25(OH)_2_D, expression of the NF-κB inhibitor IκBα was upregulated [187]. Cells infected with RSV for 24 h had decreased protein levels of IκBα, due to RSV-induced degradation, but in cells pre-treated with 1,25(OH)_2_D prior to RSV infection IκBα levels were increased due to augmented mRNA transcription and protein synthesis. An increase in IκBα results in NF-κB remaining inactive in the cytoplasm, preventing its translocation into the nucleus and subsequent binding to DNA promoter regions. Thus, vitamin D inhibits RSV induction of NF-κB-driven IFN-β and CXCL10 secretion, as also demonstrated in this study. Additionally, interferon stimulated genes (ISGs) without functional NF-κB sites in their promoters were also measured, with 1,25(OH)_2_D treatment resulting in decreased levels of human myxovirus resistance protein 1 (MxA) and ISG15. This was demonstrated to be a consequence of the suppression of NF-κB-driven IFN-β production. Similarly, pre-treatment with 1,25(OH)_2_D before inoculation with RSV resulted in decreased levels of STAT1 protein and inhibition of STAT-1 translocation to the nucleus, also as a consequence of suppression of NF-κB-driven IFN-β production. However, even though production of important components in the antiviral response to RSV infection (IFN-β, CXCL10, STAT1, MxA and ISG15) were reduced by treatment with 1,25(OH)_2_D, viral replication and viral load was not increased. Thus, via increasing IκBα levels, 1,25(OH)_2_D is able to dampen the inflammatory response to RSV infection whilst maintaining the antiviral state and without having adverse effects on viral load, suggesting a potential role in reducing immunopathology. A similar study using alveolar A549 cells infected with RSV has also demonstrated that 1,25(OH)_2_D is able to decrease levels of phosphorylated STAT-1 (pSTAT-1) and increase IκBα protein levels, with subsequent diminished expression of interferon regulatory factor 1 (IRF1), IRF7, IFN-β and CXCL8 mRNA [188]. This study also used retroviral transduction to overexpress the common M4 VDR variant, or the M1 *FokI* VDR variant, which has been associated with severe RSV bronchiolitis. No differences were observed between the two variants in viral replication, or the vitamin D-induced increase in IκBα and repression of NF-κB-regulated genes. However, in M1 *FokI* VDR-expressing cells, vitamin D was not able to repress expression of the STAT-1 regulated genes IRF1 and IRF7, due to decreased regulation of STAT-1 phosphorylation. Therefore, together these studies indicate that vitamin D is able to dampen NF-κB and STAT-1 mediated inflammation to reduce RSV immunopathology, with an impairment in the regulation of STAT-1 phosphorylation resulting in more severe disease.

It has also been demonstrated that the antimicrobial peptides LL-37 and β-defensin 2 have anti-viral activity against RSV, blocking viral cellular entry, preventing virus-induced epithelial cell death, inhibiting production of new infectious particles and diminishing the spread of infection [56,121]. Since vitamin D has been demonstrated to be able to induce secretion of both of these molecules [116,117,118], this provides a potential mechanism by which vitamin D may exert antiviral effects against RSV infection.

Finally, a number of studies have been carried out in calves, looking at the association between vitamin D and bovine RSV (BRSV). Naturally occurring BRSV infection in young ruminants has been demonstrated to mimic the pathogenesis and lesions observed in RSV infection of infants, thus providing a useful model for studying RSV infection. Conversely to the effects described above, whereby production of NF-κB-linked pro-inflammatory cytokines was inhibited, vitamin D treatment has been shown to elevate secretion of the pro-inflammatory cytokine IL-12p40 following BRSV infection [189]. No effect was observed on γδ T-cell cytokine production [190], or IFN-γ, CXCL8, TNF-α, IL-1β or IL-6 secretion [189].

### 6.3. Influenza Virus

The influenza virus is associated with seasonal epidemics, severe pandemics, pneumonia and secondary bacterial infections. As such, a simple and cost-effective prevention strategy is desirable, with clinical trial results showing promising effects of vitamin D supplementation in the prevention of seasonal influenza A infection in Japanese schoolchildren [17]. However, as with RSV and rhinovirus, *in vitro* work investigating the specific effects of vitamin D on the immune response to influenza viruses is lacking.

In a study using alveolar A549 cells, the effect of pre- or post-treatment with 1,25(OH)_2_D on infection with influenza A H1N1 was assessed [191]. It was demonstrated that vitamin D had no effect on A549 cell viability following infection with influenza virus, viral clearance or the anti-viral state. Whilst influenza infection increased the production of pro-inflammatory cytokines and chemokines, as with RSV infection [187,188], treatment with vitamin D either before or after influenza infection decreased gene expression of TNF-α, IFN-β, ISG15, CXCL8, IL-6 and RANTES (CCL5). Since an uncontrolled inflammatory response to influenza infection can cause complications such as pulmonary oedema, and the cytokine storm has been associated with more severe disease and higher mortality [192]), this suppression of the hyper-inflammatory response may be beneficial in preventing immunopathology.

The antimicrobial peptides LL-37 and human β-defensin 2 have also been shown to have anti-viral properties against influenza virus, reducing disease severity and viral replication, inhibiting infectivity, and demonstrating neutralising activity [54,193]. As described above, since vitamin D has been shown to induce production of these AMPs, this may be another mechanism by which vitamin D is able to exert antiviral effects on influenza virus infection.

Finally, the majority of work into vitamin D and influenza infection has been investigating the potential use of vitamin D as an adjuvant in influenza vaccines. However, vitamin D has not been shown to be able to modulate the humoral response to inactivated influenza virus in this setting [194,195,196,197,198].

### 6.4. Other Respiratory Viruses

In the case of other common viral causes of ARI in humans, no *in vitro* studies have been carried out to determine effects of vitamin D on the immune response to these pathogens. This is the case for human metapneumovirus, parainfluenza, adenovirus, coronaviruses, enterovirus and human bocavirus.

## 7. Conclusions

This review has identified a modest number of laboratory studies investigating immunomodulatory effects of vitamin D metabolites in respiratory epithelial cells infected with respiratory viruses. Despite clinical evidence that vitamin D supplementation may reduce susceptibility to such infections *in vivo* [28], *in vitro* studies performed to date have not shown that vitamin D metabolites inhibit viral replication in epithelial cells. However, all the studies reviewed demonstrated effects of vitamin D metabolites on expression and secretion of pro-inflammatory cytokines and chemokines. Interestingly, the effects of 1,25(OH)2D on chemokine expression and secretion varied between pathogens; this finding complements those of clinical trials suggesting that vitamin D-inducible responses may be more effective against some pathogens than others [17]. More studies are needed to characterize the *in vitro* actions of vitamin D in viral respiratory infection (particularly to investigate why suppression of type 1 interferon responses is not associated with increased viral replication), and to clarify the effects of vitamin D metabolites on respiratory virus-induced expression of cell surface markers mediating viral entry and bacterial adhesion to respiratory epithelial cells.

## Abbreviations Used

1,25(OH)_2_D: 1,25-dihydroxyvitamin D
25(OH)D: hydroxyvitamin D
7-DHC: 7-dehydrocholesterol
Ag: Antigen
AP-1: Activator protein 1
APC: Antigen presenting cell
ARI: Acute respiratory infection
CARD: Caspase recruitment domain
CCL: Cysteine-cysteine motif ligand
CD: Cluster of differentiation
CpG: Cytosine phosphate guanine
CREB: Cyclic adenosine monophosphate (cAMP) responsive element-binding protein
CXCL: Cysteine-X-cysteine motif ligand
CYP: cytochrome p450
DC: Dendritic cell
ds: Double-stranded
FADD: FAS associated death domain
GAS: IFN-γ activated site
HBD2: Human beta defensin 2
IFN: Interferon
IFNAR1: IFN alpha receptor 1
Ig: immunoglobulin
IκBα: Nuclear factor of kappa light polypeptide gene enhancer in B-cells inhibitor alpha
IKK: Inhibitor of NF-κB kinase
IL: Interleukin
iNOS: Inducible nitric oxide synthase
IPS-1: IFN-β promoter stimulator 1
IRAK: Interleukin-1-receptor-associated kinase 1
IRF: Interferon regulatory factor
ISG: Interferon stimulated gene
ISGF3: interferon-stimulated gene factor 3
ISRE: interferon stimulated response element
JAK: Janus kinase
JNK: c-jun N-terminal kinase
LRR: Leucine rich repeat
MAPK: Mitogen activated protein kinase
MDA5: Melanoma differentiation-associated protein kinase
MEK: mitogen-activated protein kinase kinase
MHC: Major histocompatibility complex
MxA: Myxovirus resistance protein 1
MYD88: Myeloid differentiation primary response 88
NAP: NF-κB activating kinase (NAK)-associated protein
NEMO: NF-κB essential modulator
NF-κB: nuclear factor kappa B
NOD: Nucleotide-binding oligomerisation domain
PAMP: pathogen-associated molecular pattern
PRR: pattern recognition receptor
Raf: Mitogen-activated protein kinase kinase kinase
Ras: Mitogen-activated protein kinase kinase kinase kinase
RIG-1: Retinoic acid-inducible gene-1
RIP1: Receptor interacting protein
RNA Pol II: RNA polymerase II complex
ROS: Reactive oxygen species
RSV: Respiratory syncytial virus
RV: Rhinovirus
RXR: Retinoid X receptor
SINTBAD: Similar to NAP-1 TBK adaptor
ss: Single-stranded
STAT: signal transducer and activator of transcription
TAB: TGF-β activated kinase
TAK: TGF-β activated kinase
TANK: TRAF family member associated NF-κB activator
TBK: TANK-binding kinase
TCR: T-cell receptor
TGF: Transforming growth factor
TIR: Toll/IL-1 receptor
TLR: Toll-like receptor
TNF: tumour necrosis factor
TRADD: TNF receptor associated death domain
TRAF: TNF receptor associated factor
Treg: Regulatory T-cell
TRIF: TIR-containing adaptor inducing IFN-β
TYK: tyrosine kinase
VRD: vitamin D receptor
VDRE: vitamin D responsive element. Circled P’s in figures represent phosphorylation
